# Cannabis use disorder, suicide attempts, and self-harm among adolescents: A national inpatient study across the United States

**DOI:** 10.1371/journal.pone.0292922

**Published:** 2023-10-17

**Authors:** Adeolu Funso Oladunjoye, Elijah Li, Kammarauche Aneni, Edore Onigu-Otite

**Affiliations:** 1 Menninger Department of Psychiatry, Baylor College of Medicine, Houston, Texas, United States of America; 2 School of Medicine, Baylor College of Medicine, Houston, Texas, United States of America; 3 Child Study Center, Yale School of Medicine, New Haven, Connecticut, United States of America; Duke University Medical Center: Duke University Hospital, UNITED STATES

## Abstract

**Background:**

Suicide is among the top three causes of adolescent mortality. There is a scarcity of research examining cannabis use and suicidal behavior in adolescents.

**Objectives:**

To determine the association between cannabis use disorder (CUD) and suicide attempt/self-harm in a hospitalized sample of adolescents.

**Methods:**

We conducted a cross-sectional observation study using data from the Nationwide Inpatient Sample collected over four years from January 1, 2016, through December 31, 2019. We included adolescents aged 10–19 hospitalized during the above period (N = 807,105). The primary outcome was suicide attempt/self-harm and the main predictor was CUD. The International Classification of Diseases Tenth Revision (ICD 10) diagnostic codes was used to identify a diagnosis of CUD, suicide attempt/self-harm, and other diagnoses included in the analyses. Adolescents diagnosed with CUD (n = 53,751) were compared to adolescents without CUD (n = 753,354). Univariate and multivariate logistic regressions were conducted to determine the association between CUD and suicide attempts/self-harm.

**Results:**

807,105 adolescent hospitalizations were analyzed, of which 6.9% had CUD. Adolescents with CUD were more likely to be older (17 years vs. 15 years), female (52% vs. 48%), have depression (44% vs. 17%), anxiety (32% vs. 13%), an eating disorder (1.9% vs. 1.2%), ADHD (16.3% vs. 9.1%), Conduct Disorder (4.1% vs. 1.3%), Alcohol Use Disorder (11.9% vs. 0.8%), Nicotine Use Disorder (31.1% vs. 4.1%), Cocaine Use Disorder (5.4% vs. 0.2%), Stimulant Use Disorder (0.8% vs. 0.4%) and report suicide attempts/self-harm (2.8% vs. 0.9%) [all ps<0.001]. After adjusting for potential confounders, CUD was associated with a higher risk of suicide attempts/self-harm (OR = 1.4, 95% CI 1.3–1.6, p <0.001).

Post-hoc analyses showed the presence of depression moderated the association between CUD and suicide attempts/self-harm in that adolescents with CUD and depression had 2.4 times the odds of suicide attempt/self-harm compared to those with CUD but no depression after controlling for potential confounders (p<0.001).

**Conclusions:**

Our study provides evidence for the association between CUD and suicide risk among hospitalized adolescents and underscores the importance of recognizing and addressing co-occurring mental and substance use disorders along with CUD to mitigate suicide risk. Identifying high-risk adolescents in inpatient settings provides an opportunity for intervention.

## Introduction

Suicide is one of the top three leading causes of adolescent death, accounting for about 1–2 in 10 deaths among 12–19-year-olds [1,2]. The history of a suicide attempt is one of the strongest predictors of completed suicide in adolescents [3,4]. The prevalence of suicide, suicide planning, suicide attempts, and suicide-related hospitalizations are rising among adolescents [5,6]. These concerning statistics underscore the importance of identifying risk factors for suicidal behaviors to develop and implement effective solutions.

Substance use is associated with an increased risk of engaging in suicidal behaviors [7,8]. Cannabis is the most used illegal substance worldwide and in the United States. In 2019, 18% of Americans reported using cannabis at least once [9]. In 2019, 37% of US high school students reported lifetime use of marijuana, and 22% reported use in the past 30 days [10]. Almost one in five adolescents had used cannabis frequently between ages 13 and 17 (26.6% of males, 9.8% of females) [11]. The legalization of cannabis in some states has contributed to a lower risk-perception including among youth [12]. Coupled with increasing availability, the rise in cannabis use among adolescents and young adults is concerning in light of evidence that adolescents are susceptible to its harmful effects on brain development and neurocognitive functioning [13,14]. In some studies, evidence suggests that at this stage of development, the brain is exposed to effects that directly impact behavioral changes, including suicidal behavior [15–17].

Adolescence is a critical period for neurocognitive, emotional, psychological, and social development, with the various processes involved extending into early adulthood [18]. Animal studies demonstrate that cannabis exposure in adolescence alters gene expression associated with the structural maturation of neuronal cells in the prefrontal cortex and with reward and stress reactivity in the amygdala [19,20]. Alteration in the endocannabinoid system with early cannabis use can result in persisting neurological effects leading to poor cognitive and emotional outcomes lasting into adulthood [21]. Human studies suggest that at this stage of neurodevelopment, exposure to the effects of exogenous cannabinoids directly impacts behavioral changes, including suicidal behavior [15–17]. Recent evidence suggests the activation of the CB1 receptor by exogenous cannabinoids can diminish the production of neuronal growth factor and affect other signaling cascades involved in synapsis formation [16]. Some studies have found that cannabis exposure during adolescence is associated with altered cerebral cortical development increasing the risk of neurocognitive problems such as memory, learning, persisting attentional deficits, and impulse control problems. Such changes in neurocognitive function due to cannabis use may increase the risk of occurrence or worsening of other mental health symptoms [13]. This in turn, may increase the risk of maladaptive behaviors, which prevent individuals from adapting to the changes in various aspects of life that come with adolescence [16]. Such challenges can increase the risk of stress, depression, and anxiety, increasing the risk of suicidal behaviors [15].

Most studies that examined the association between substance use and suicidal behavior in adolescents have not focused on cannabis, and available studies have been predominantly conducted in the adult population [15,22–25]. Existing studies examining the association between cannabis use and suicidal behaviors among adolescents have focused on the general population [26,27] or included a small inpatient population limited to adolescents admitted for psychiatric hospitalization [28,29]. Adolescents, including those with ongoing cannabis use, most often present for care in medical settings for medical problems and may not be seen by a psychiatrist in these settings unless a consult is requested and a provider is available, an increasing challenge in context of mental health provider shortage. Individuals with cannabis use are often hospitalized for medical reasons, including some due to complications of cannabis use [30–32]. Additionally, due to a national shortage of psychiatric beds, adolescents with mental health problems including CUD are increasingly hospitalized on non-psychiatric pediatric beds [33]. Therefore, this study builds on existing studies using a nationally representative adolescent inpatient that includes both a psychiatric and non-psychiatric sample spanning four years and aims to examine the association between Cannabis use disorder (CUD) and suicidal attempt/self-harming behavior among adolescent hospitalizations.

We hypothesized that among hospitalized adolescents, adolescents with CUD would have a higher likelihood of suicide attempts/self-harm behaviors compared to adolescents without CUD. Understanding the association between cannabis use disorder and suicidal behaviors among adolescents in the inpatient setting will expand our understanding of substance use risk factors for suicidal behaviors among hospitalized adolescents and inform targeted interventions that can be implemented in the inpatient setting.

## Materials and methods

### Study design and data sources

A cross-sectional study was conducted based on the Nationwide Inpatient Sample (NIS) data administered by the Agency for Healthcare Research and Quality, a part of the Healthcare Cost and Utilization Project (HCUP) [34]. The NIS is the largest publicly available all-payer inpatient care database designed to produce U.S. regional and national estimates of inpatient utilization, access, cost, quality, and outcomes. The study period began in 2016, the first full year the International Classification of Diseases Tenth Revision Clinical Modification (ICD-10-CM) billing codes were available in the NIS database up until 2019. All adolescent (10–19 years) admissions from January 1, 2016, through December 31, 2019, were included in the analyses using the World Health Organization age definition of adolescents defined as the phase of life between child and adulthood from ages 10 to 19. Since the database is de-identified and publicly available, ethical clearance or Institutional Review Board approval was not required. Also, authors had no access to any information that could identify individual participants during or after data collection.

### Study population and characterization of variables

All adolescent inpatient hospitalizations (ages 10–19) from the NIS database between 2016 and 2019 were included totaling about 807,105. Within this sample, we compared adolescents admitted with an ICD-10 diagnosis of CUD to adolescents without, regardless of whether CUD was the primary or comorbid diagnosis. Similarly, we compared adolescents admitted with ICD-10 codes of suicide attempt/self-harm to those without. We also identified other co-occurring conditions in these adolescent age groups, including diagnosis of depression, anxiety, eating disorder, Attention Deficit Hyperactivity Disorder (ADHD), Intellectual and Developmental Disabilities (IDD), Conduct disorder, Substance Use Disorders (Alcohol, Nicotine, Cocaine and Stimulants).

### Patient demographics and comorbidity characteristics

Patient-level characteristics from the database included age (10–19 years), race (white, Black, Hispanic, Asian, and others), primary payer (public, private, and self-pay), regions of the US (northeast, south, mid-west/north central, and west), the All Patients Refined Diagnosis Related Groups (APRDRG) severity of illness score (minor, moderate, major and extreme) and discharge disposition (home, short term facility, skilled nursing home, home health care, discharge against medical advice (AMA) and died). Co-occurring conditions included depression, anxiety, and eating disorder, ADHD, IDD, Conduct disorder, Substance Use Disorders (Alcohol, Nicotine, Cocaine and Stimulants).

### Statistical analysis

The prevalence of cannabis use disorder and suicide attempt/self-harm were determined in this adolescent population from 2016–2019. Demographic and clinical characteristics of hospitalizations with CUD were compared to hospitalizations without CUD. Analyses used only non-missing data, constituting less than 10% of the sample. We conducted univariate and multivariate logistic regression analyses to determine the association between CUD and suicide attempt/self-harm. In addition to this first model that combined suicide attempt/self-harm into a single outcome, we conducted separate analyses with suicide attempt and self-harm as two separate outcomes to determine if the association with CUD differed by each outcome. Multivariable logistic regression analyses were adjusted for independent factors, including age, sex, race, primary payer, the severity of illness score, and illness comorbidities, including depression, anxiety, eating disorder, ADHD, IDD, Conduct disorder, Substance Use Disorders (Alcohol, Nicotine, Cocaine and Stimulants). Post-hoc analyses was conducted to explore the moderating effect of depression and anxiety on the association between CUD and suicide attempt/self-harm. We focused on depression and anxiety as these are the two most common mental disorders in adolescence. STATA version 15.0 (College Station, TX) was used for all statistical analyses. A P-value of <0.05 and a 95% confidence interval (CI) was used in the analysis.

### Results

Table 1 describes the demographic and clinical characteristics of adolescents in the total sample and in adolescents with and without CUD. The prevalence of CUD in this population was found to be about 6.9%. The overall mean age was 16 years. Adolescents in the CUD group were more likely to be older (17 years vs. 15 years, p<0.001), more likely to be females (52% vs. 48%, p<0.001), more likely to be diagnosed with depression (44% vs. 17%, p<0.001), anxiety (32% vs. 13%, p<0.001), an eating disorder (1.9% vs. 1.2%, p<0.001), ADHD (16.3% vs. 9.1%, p<0.001), Conduct Disorder (4.1% vs. 1.3%), and suicide attempts (2.8% vs. 0.9%, p<0.001). Adolescents with CUD were also more likely to have Alcohol Use Disorder (11.9% vs. 0.8%, p<0.001), Nicotine Use Disorder (31.1% vs. 4.1%, p<0.001), Cocaine Use Disorder (5.4% vs. 0.2%, p<0.001) and Stimulant Use Disorder (0.8% vs. 0.4%, p<0.001). Over half of the adolescents in the CUD group were white (57%) and had a moderate APRDRG severity (51%). Insurance coverage varied, with public insurance (55.0%) being the predominant insurance coverage, followed by private insurance (41.4%) and self-pay 3.6%. Most patients were from the southern part of the country, with an overall proportion of about 40.1% (p<0.001). We dropped 76,646 participants from the dataset with missing values among the variables of interest, given that the proportion of missing data was less than 10% and, therefore, unlikely to impact the internal validity of our findings. Missing data included: sex (n = 133), race (n = 44,821), insurance (n = 32,848), APRDRG status (n = 975), and disposition on discharge (n = 603).

**Table 1:** Patient demographics and clinical characteristics of adolescent hospitalizations with cannabis use disorder.

Variable name	All (n = 807,105)	Study Groups		
		Non- CUD group (n = 753,354)	CUD group (n = 53,751)	*P Value*
**Mean Age (±SE)**	15.82 ± 0.00	15.73± 0.00	17.20 ± 0.01	<0.0001
**Age category**				
**10–14**	30.7	32.3	8.0	
**15–19**	69.3	67.7	92.0	<0.0001
**Sex**				
**Female**	62.2	62.9	51.6	
**Male**	37.8	37.1	48.4	<0.0001
**Race, %**				
**White**	51.0	50.6	56.9	
**Black**	19.2	19.1	20.4	
**Hispanic**	21.5	22.0	15.2	
**Asian**	2.5	2.5	1.5	
**Others**	5.8	5.8	6.0	<0.0001
**Insurance, %**				
**Public**	55.0	55.1	53.5	
**Private**	41.4	41.4	40.8	
**Self- Pay**	3.6	3.5	5.7	<0.0001
**Region, %**				
**Northeast**	16.1	16.0	17.4	
**Mid-West/North Central**	23.4	23.0	30.0	
**South**	40.1	40.5	33.6	
**West**	20.4	20.5	19.0	<0.0001
**APRDG severity**				
**Minor**	40.5	40.8	35.6	
**Moderate**	41.8	41.2	51.2	
**Major**	14.0	14.2	11.0	
**Extreme**	3.7	3.8	2.2	<0.0001
**CUD as primary diagnosis**	0.1	0.0	2.1	<0.0001
**Co-morbidities**				
**Depression**	18.7	16.9	43.9	<0.0001
**Anxiety**	14.5	13.3	31.8	<0.0001
**Eating Disorder**	1.2	1.2	1.9	<0.0001
**ADHD**	9.6	9.1	16.3	<0.0001
**Conduct disorder**	1.5	1.3	4.1	<0.0001
**IDD**	1.3	1.3	0.4	<0.0001
**Other Substance use disorders**				
**Alcohol use disorder**	1.5	0.8	11.9	<0.0001
**Nicotine use disorder**	5.9	4.1	31.1	<0.0001
**Cocaine use disorder**	0.5	0.2	5.4	<0.0001
**Stimulant use disorder**	0.8	0.4	0.8	<0.0001
**Suicide Attempt/Self-harm**				
**Yes**	1.0	0.9	2.8	
**No**	99.0	99.1	97.2	<0.0001
**Disposition on discharge**				
**Home**	91.8	92.0	88.7	
**Short term facility**	1.3	1.3	1.3	
**Skilled nursing home**	3.7	3.4	7.1	
**Home health care**	2.2	2.4	0.9	
**Discharge AMA**	0.7	0.6	1.9	
**Died**	0.3	0.3	0.1	<0.0001

n: Sample number; SE: Standard error; %: Percentage; CUD–Cannabis use disorder.

#### Factors associated with CUD and suicide attempt/self-harm in adolescent hospitalizations

In univariate logistic regression, having a CUD diagnosis increased the odds of having a diagnosis of suicide attempt/self-harm by 218%. In a multivariate logistic regression model after controlling for age, sex, race, insurance, APRDRG severity, and other co-occurring disorders (Depression, Anxiety, Eating Disorder, ADHD, IDD, Conduct Disorder, Alcohol Use Disorder, Nicotine Use Disorder, Cocaine Use Disorder, Stimulant Use Disorder), adolescents with a diagnosis of CUD had 40% higher odds of being engaged in suicide attempt/self-harm (95% CI: 1.32–1.55, p<0.001) (Table 2). As noted above, we also conducted analysis with suicide attempts and self-harm as separate outcomes (See S1 Table). In the multivariate analysis, CUD was associated with 40% higher odds of suicide attempts and 40% higher odds of self-harm (S1 Table).

**Table 2:** Factors associated with Cannabis use disorder and suicide attempt/self-harm in adolescent hospitalizations.

Variable name	Univariate analysis (Unadjusted OR)	Multivariate analysis (Adjusted OR)
**Mean Age (±SE)**	1.00 (0.99–1.01)	0.98 (0.97–0.99) ***
**Age category**		
**10–14**	Reference	
**15–19**	1.05 (0.99–1.12)	0.89 (0.84–0.95) ***
**Cannabis use disorder**		
**No**	Reference	Reference
**Yes**	3.18 (2.97–3.41) ***	1.43 (1.32–1.55) ***
**Sex**		
**Male**	Reference	Reference
**Female**	1.29 (1.22–1.36) ***	1.18 (1.11–1.25) **
**Race, %**		
**White**	Reference	Reference
**Black**	0.49 (0.46–0.54) ***	0.71 (0.65–0.77) ***
**Hispanic**	0.50 (0.45–0.57) ***	0.78 (0.69–0.87) ***
**Asian**	0.76 (0.63–0.91) **	1.04 (0.87–1.25)
**Others**	0.80 (0.71–0.91) **	0.95 (0.84–1.07)
**Insurance, %**		
**Public**	Reference	Reference
**Private**	1.30 (1.21–1.39) ***	1.02 (0.95–1.10)
**Self- Pay**	1.50 (1.31–1.74) ***	1.40 (1.22–1.61) ***
**APRDG severity**		
**Minor**	Reference	Reference
**Moderate**	1.45 (1.37–1.54) ***	1.07 (1.01–1.14) **
**Major**	0.94 (0.86–1.03)	0.88 (0.80–0.97) *
**Extreme**	1.17 (0.01–1.34) *	1.45 (1.26–1.68) ***
**Co-morbidities**		
**Depression**	9.55 (8.91–10.22) ***	7.06 (6.52–7.66) ***
**Anxiety**	4.09 (3.82–4.37) ***	1.32 (1.22–1.42) ***
**Eating Disorder**	3.78 (3.36–4.25) ***	1.45 (1.29–1.64) ***
**ADHD**	2.32 (2.18–2.47) ***	1.41 (1.32–1.50) ***
**IDD**	0.92 (0.74–1.14) **	1.40 (1.12–1.76) **
**Conduct disorder**	2.16 (1.88–2.48) **	1.32 (1.12–1.55) **
**Substance use disorders**		
**Alcohol use disorder**	3.96 (3.57–4.40) ***	1.53 (1.36–1.72) ***
**Nicotine Use disorder**	2.81 (2.60–3.03) ***	1.48 (1.36–1.62) ***
**Cocaine Use disorder**	3.07 (2.55–3.70) ***	1.14 (0.93–1.40)
**Stimulant Use disorder**	3.01 (2.60–3.51) ***	1.27 (1.08–1.50) ***

SE: Standard error, %: Percentage; Ref–reference group

*<0.05

**<0.01

***<0.001.

We found that compared to adolescents with no CUD and no depression, after controlling for potential confounders, higher odds of suicide attempt/self-harm occurred in adolescents who had no CUD with depression (OR:8.6, 95% CI: 7.9, 9.4), CUD with no depression (3.8, 95% CI: 3.4, 4.4) and CUD with depression (OR: 9.0, 95% CI: 8.0, 10.2) (Table 3). Likewise, compared to adolescents with no CUD and no anxiety, after controlling for potential confounders, higher odds of suicide attempts/self-harm was found in adolescents with no CUD and anxiety (OR: 1.5, 95% CI: 1.4, 1.6), CUD with no anxiety (OR: 1.8, 95% CI: 1.6, 2.0), CUD with anxiety (OR: 1.6, 95% CI: 1.4, 1.8) (Table 3).

**Table 3:** Cannabis use disorder, depression, anxiety, and suicide attempt/self-harm in adolescent hospitalizations.

Study Group	Variable name	Univariate analysis (Unadjusted OR)	Multivariate analysis (Adjusted OR)
CUD*Depression	**No CUD + No Depression**	reference	reference
	**NO CUD + Depression**	10.81 (10.07–11.60) ***	8.62 (7.93–9.38) ***
	**CUD + No Depression**	4.72 (4.23–5.27) ***	3.83 (3.38–4.35) ***
	**CUD + Depression**	13.04 (11.80–14.41) ***	9.04 (8.02–10.20) ***
CUD*Anxiety	**No CUD + No Anxiety**	reference	reference
	**NO CUD + Anxiety**	4.43 (4.13–4.76) ***	1.46 (1.34–1.59) ***
	**CUD + No Anxiety**	3.85 (3.53–4.19) ***	1.79 (1.62–1.98) ***
	**CUD + Anxiety**	6.25 (5.62–6.96) ***	1.62 (1.43–1.82) ***

SE: Standard error, %: Percentage; Ref–reference group;

*<0.05,

**<0.01,

***<0.001.

**Fig 1:**
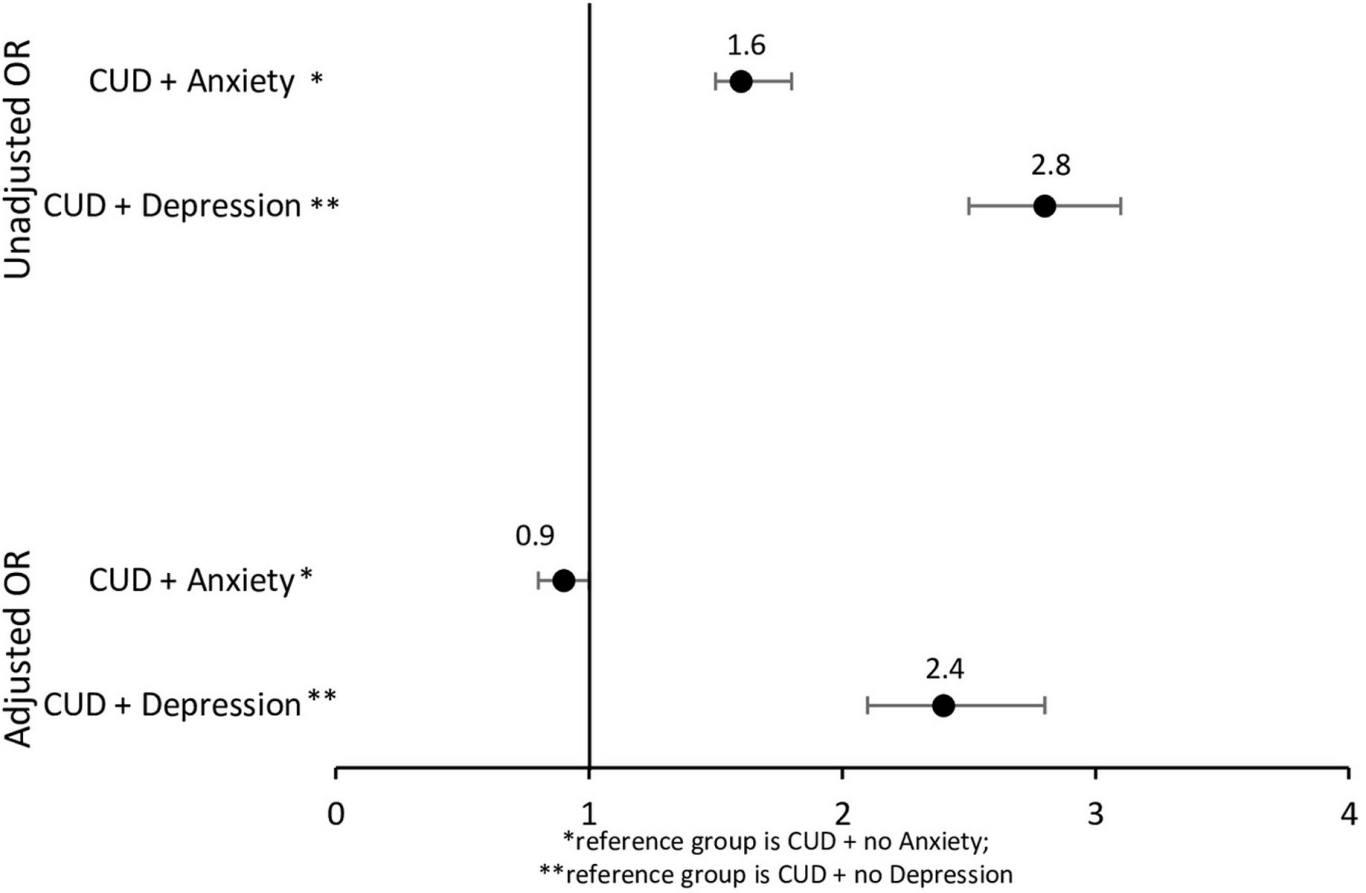
Moderation analyses: Odds of suicide attempt/self-harm among adolescents with CUD and depression or anxiety compared to Adolescents with CUD but no depression or anxiety.

When we examined the moderating effect of depression on the association between CUD and suicide risk, we found that the presence of depression moderated this association such that those with CUD and depression had 2.4 times the odds of suicide risk compared to those with CUD but no depression after controlling for potential confounders (p<0.001) (Fig 1). Likewise, adolescents with CUD and anxiety had 1.6 times the odds of suicide risk compared to adolescents with CUD but no anxiety (p<0.001) (Fig 1). However, this association was lost after controlling for potential confounders (p>0.05) (Fig 1).

## Discussion

We conducted a study to determine the association between cannabis use disorder (CUD) and suicidal attempt/self-harm using a nationally representative inpatient adolescent sample. The present study found that CUD was strongly associated with suicidal attempts/self-harm both as a combined outcome and separate outcomes (suicide attempt and self-harm) after controlling for potential confounders. This finding is consistent with previous non-inpatient epidemiological studies [35,36], and a prior adolescent inpatient psychiatric sample [29], but in contrast to the adolescent inpatient study by Sellers et al., which found no independent association between cannabis use and self-harm [28], possibly due to the small sample size.

Findings from our study suggest an independent association between CUD and suicidal behaviors and non-suicidal self-harm and underscore the importance of addressing CUD among adolescents in inpatient settings. Clinically, while non-accidental self-harm may be clear, the degree of suicidal intent behind such actions can be more obscure. Since our study is cross-sectional, we cannot determine whether CUD preceded suicidal behaviors or vice-versa. Nevertheless, the inpatient setting provides a unique opportunity to engage adolescents in addressing cannabis use regardless of their reason for hospitalization, particularly those adolescents who may not otherwise have been involved in treatment prior to the hospitalization. The inpatient setting may also lend itself to automated, evidence-based digital interventions that the adolescent can access without the need for additional provider manpower. However, potential limitations in implementing these interventions can arise from limited or no access to digital devices while on the inpatient unit, the severity of illness that may hinder the adolescent ability to engage in self-guided digital interventions. However, the inpatient setting can allow for the identification of high-risk adolescents who require follow-up substance use treatment. The inpatient setting also provides an opportunity to implement multidisciplinary approaches that leverage healthcare providers, families, and community resources to develop a comprehensive plan to support the adolescent. Future studies are needed to investigate inpatient approaches to inform specific intervention strategies.

Our study also found that depression, anxiety, eating disorders, ADHD, Conduct Disorder, Alcohol Use Disorder, Nicotine Use Disorder, Cocaine Use Disorder and Stimulant Use Disorder were significantly associated with CUD. In addition, except for Cocaine Use Disorder, these factors were also strongly associated with suicidal attempts/self-harm independent of CUD. Furthermore, in the analyses that examined suicide attempts and self-harm as separate outcomes, depression, anxiety, ADHD, and stimulant use disorder were associated with suicide attempts and self-harm independent of CUD. Although this is a cross-sectional study which cannot establish causation, other studies have shown that cannabis use increases the risk for developing other illicit drug use (the gateway hypothesis) [37] as well as other mental disorders during adolescence [38,39], specifically depression and anxiety, which can then further increase the risk of self-harm or suicide [15]. Our study revealed that hospitalized adolescents with CUD had over double the rates of depression, anxiety, ADHD, Conduct Disorder, and all the substance use disorders included (Alcohol, Nicotine, Cocaine and Stimulant) when compared to adolescents without CUD. We also found that comorbid CUD and depression were associated with higher odds of suicidal behavior compared to CUD alone, similar to findings from a recent publication [27]. Both depression and anxiety, being internalizing disorders, are often chronic and less readily detected. A prospective cohort study lasting seven years found that frequent cannabis use in teenage girls predicted later depression and anxiety, with daily users carrying the highest risk [40]. Similarly, a systematic review and meta-analysis found that that cannabis use in adolescence increased the risk of developing depression and anxiety in young adulthood [41]. These findings highlight the critical importance of a thorough mental health and substance use history when assessing adolescents in the inpatient setting and at all patient encounters, including in primary care and emergency room settings. In addition, our findings underscore the importance of the dual diagnosis approach of addressing both substance use and co-occurring mental disorders to alleviate suicide risk among adolescents [42,43].

In contrast to a prior nationally representative study among non-inpatient adolescents which showed no racial/ethnic differences in the association between cannabis use and suicidal risk [44], our study also revealed lower odds of suicidal/self-harm behaviors among Black and Hispanic adolescents compared to white adolescents. These lower odds appeared to be in relation to the risk for self-harm and not suicide attempts (See S1 Table) in contrast to more recent studies showing higher rates of suicide attempts among Black adolescents compared to the white adolescents [45,46]. Although, historical data shows comparatively lower rates of suicide among Black and Hispanic adolescents, our finding of no difference in the risk for suicide attempts between Black and white adolescents, would be consistent with the alarming rise in suicide rates among Black adolescents [47]. Regarding self-harm, studies investigating racial/ethnic differences in non-suicidal self-harm have revealed mixed findings [48]. However, a recent systematic review found higher prevalence among non-Hispanic white adolescents and specifically Black boys [48]. Future studies should further clarify these findings, investigate potential racial and sex differences and investigate potential reasons for these differences.

Additional outcomes of this study suggest that female sex, being from the US Mid-west/North Central or South, having a self-pay payment option, and having co-occurring psychiatric disorders are associated with CUD and suicide attempt/self-harm among adolescents’ hospitalizations. It is plausible that having a self-pay payment option may reflect a higher socioeconomic status and the ability to seek and obtain healthcare. Other studies are unclear about the relationships between cannabis use, CUD and suicidal attempts or self-harm, and other variables such as sex, insurance type, and location [49,50]. Although the relationship remains poorly understood, consistent with our study, other studies find that females are more susceptible to adverse outcomes after cannabis use [42,51,52]. However, some studies report that men consume more cannabis than by women and this consumption is associated with co-occurring suicide behaviors [53,54]. When we separated out the outcomes, female sex was associated with self-harm but not suicide attempts. This difference may relate to findings indicating that females are more likely to report non-suicide self-injury behaviors than men [55]. Future studies are needed to investigate these findings.

### Strengths and limitations

The present study contributes to the literature on a subject that needs to be vitally addressed by looking at a large sample size of nationally representative hospitalized adolescents. Including adolescents with CUD in non-psychiatric beds is a unique feature of this study since many adolescents with CUD are admitted for non-psychiatric reasons and/or admitted to non-psychiatric beds. The findings of the present study should be interpreted in the context of possible limitations. As with any cross-sectional study, our study cannot establish any causative effects between cannabis use disorder, suicidal behaviors, depression, or anxiety. While the present study elucidates various associations between CUD and suicide and self-harm in adolescent hospitalizations, no causal conclusions can be drawn. Since this study included both psychiatric and non-psychiatric hospitalizations, we cannot determine if our findings are applicable to adolescents admitted for psychiatric hospitalizations versus not which might have implications for developing and administering interventions to mitigate suicide risk in the inpatient setting. In addition, our findings are not generalizable to non-inpatient samples. Given that diagnosis may be inconsistently reported and document, CUD may be underdiagnosed in the current sample which would result in under-estimation of associations.

The lack of assessment of severity of CUD is a limitation as it may be reasonable to think that adolescents with heavier cannabis use may experience more mental health problems resulting in increased rates of self-harm or suicide. Those with more severe CUD may also have higher rates of depression and anxiety. Although it was not the primary focus of this study, the lack of assessment of the severity of depression is a limitation given that adolescents with more severe depression may likely have more suicide attempts or self-harm. The measurement of suicidality in adolescents also constitutes a limitation. The intent behind suicidal behaviors is often unclear, sometimes even to the adolescents involved and it is sometimes difficult to confirm if or when self-injury was intended for death, leaving the intent undetermined [56,57]. As much as we intended to include those with intentional suicidal self-injury, it is possible that some of our study population included those who experienced non- suicidal self-injury (NSSI). However, what is clear is that NSSI is associated with a higher risk of suicide attempts and suicide [58–60]. Regardless of these limitations, our findings underscore the importance of assessing suicide risk in patients admitted with CUD and highlight the need to undertake a dual diagnosis approach to mitigate suicide risk among adolescents.

### Conclusion

To our knowledge, this is the first study examining the association between cannabis use and suicidal attempts or self-harm in a nationally representative adolescent hospital sample. The results of this study indicate that suicide attempts and self-harm are associated with CUD in hospitalized adolescents and that this association is moderated by depression. Identifying high-risk adolescents during hospitalizations, regardless of their reason for admission, and initiating early interventions at this point of care with subsequent follow-up care and support should be a key component of comprehensive suicide prevention strategies in this vulnerable population.

Our study revealed high levels of co-occurring depression, anxiety, ADHD, Alcohol Use Disorder and Nicotine Use Disorder in adolescents with CUD. Depression and anxiety, both being internalizing disorders, may be poorly recognized or undertreated leaving adolescents dealing with their symptoms in unhealthy ways, including by using cannabis, further worsening their condition. Most adolescents, with mental health and substance use problems including those with ongoing cannabis use, do not present to psychiatrists, who, as a specialty, are in very short supply. Rather, most adolescents with ongoing cannabis use most often present to non-psychiatric visits such as to annual physical, visits with primary care, or emergency care. Early intervention including identification and optimal treatment of comorbid mental and substance use disorders could result in reduction of symptoms, potentially reduce the risk of progression to self-harming behavior. In adolescents with CUD, the dual diagnosis approach may result in more robust patient outcomes.

With the recent rise in Cannabis use among adolescents, and the increased risk of mental health problems including self-harm/suicide, findings from this study underscore the need for targeted prevention efforts that address cannabis use among adolescents in inpatient settings. Our study highlights the need for policies that promote adolescent mental health education and limit access to cannabis use during adolescence which remains a vulnerable neurodevelopment stage.

## Supporting information

S1 Table Multivariate analysis testing association with Cannabis use disorder (CUD) and suicide attempt / CUD & self-harm in adolescent hospitalizations. (DOCX)

S2 Table ICD 10 codes for diagnosis used in this study. (DOCX)
