# Correction: Cannabis use disorder, suicide attempts, and self-harm among adolescents: A national inpatient study across the United States

**DOI:** 10.1371/journal.pone.0294303

**Published:** 2023-11-30

**Authors:** The PLOS ONE Staff

## Notice of republication

This article was republished on October 24, 2023, to correct the error in the title that was introduced during the typesetting process. The publisher apologizes for the error. Please download this article again to view the correct version. The originally published, uncorrected article and the republished, corrected articles are provided here for reference.

## Supporting information

S1 File Originally published, uncorrected article. (PDF)

S2 File Republished, corrected article. (PDF)
