# Supplementary material for: Cannabis use disorder, suicide attempts, and self-harm among adolescents: A national inpatient study across the United States
Source: PLoS One. 2023 Oct 17;18(10):e0292922. doi: 10.1371/journal.pone.0292922 (PMC10581466; doi:10.1371/journal.pone.0292922)
Supplement: S1 Table — (DOCX) [file pone.0292922.s001.docx]

**S1 Table: Multivariate analysis testing association with Cannabis use disorder (CUD) and suicide attempt / CUD & self-harm in adolescent hospitalizations**

| **Variable name** | **CUD & Suicide attempt**  **Multivariate analysis (Adjusted OR)** | **CUD & Self-harm**  **Multivariate analysis (Adjusted OR)** |
| --- | --- | --- |
| **Age category** |  |  |
| **10-14** | Reference |  |
| **15-19** | 1.0 (0.8-1.1) | 0.9 (0.8-0.9) *** |
| **Cannabis use disorder** |  |  |
| **No** | Reference | Reference |
| **Yes** | 1.4 (1.2-1.7) *** | 1.4 (1.3-1.5) *** |
| **Sex** |  |  |
| **Male** | Reference | Reference |
| **Female** | 1.1 (0.9-1.2) | 1.2 (1.1-1.2) *** |
| **Race, %** |  |  |
| **White** | Reference | Reference |
| **Black** | 1.1 (0.9-1.3) | 0.7 (0.6-0.7) *** |
| **Hispanic** | 1.0 (0.8-1.3) | 0.8 (0.7-0.8) *** |
| **Asian** | 1.4 (1.0-2.1) | 1.0 (0.8-1.2) |
| **Others** | 1.4 (1.1-1.8) * | 0.9 (0.8-1.0) |
| **Insurance, %** |  |  |
| **Public** | Reference | Reference |
| **Private** | 1.1 (1.0-1.3) | 1.0 (0.9-1.1) |
| **Self- Pay** | 1.8 (1.3-2.4) *** | 1.3 (1.2-1.6) *** |
| **APRDG severity** |  |  |
| **Minor** | Reference | Reference |
| **Moderate** | 0.8 (0.7-0.9) *** | 1.1 (1.1-1.2) *** |
| **Major** | 0.7 (0.6-0.9) ** | 0.9 (0.8-1.0) * |
| **Extreme** | 0.9 (0.6-1.4) | 1.5 (1.3-1.7) |
| **Co-morbidities** |  |  |
| **Depression** | 9.4 (7.8-11.1) *** | 6.8 (6.2-7.4) *** |
| **Anxiety** | 1.3 (1.1-1.6) ** | 1.3 (1.2-1.4) *** |
| **Eating Disorder** | 1.0 (0.7-1.5) | 1.5 (1.3-1.7) *** |
| **ADHD** | 1.4 (1.2-1.6) *** | 1.4 (1.3-1.5) *** |
| **IDD** | 1.1 (0.6-2.1) | 1.4 (1.1-1.8) ** |
| **Conduct disorder** | 1.0 (0.7-1.5) | 1.4 (1.2-1.6) *** |
| **Substance use disorders** |  |  |
| **Alcohol use disorder** | 1.3 (1.0-1.8) | 1.6 (1.4-1.8) *** |
| **Nicotine use disorder** | 1.2 (1.0-1.5) | 1.5 (1.4-1.7) *** |
| **Cocaine use disorder** | 0.8 (0.4-1.3) | 1.1 (0.9-1.4) |
| **Stimulant use disorder** | 1.9 (1.3-2.8) *** | 1.2 (1.0-1.5) * |

SE: Standard error, %: percentage; Ref – reference group; *<0.05, **<0.01, ***<0.001
