# Supplementary material for: Cannabis use disorder, suicide attempts, and self-harm among adolescents: A national inpatient study across the United States
Source: PLoS One. 2023 Oct 17;18(10):e0292922. doi: 10.1371/journal.pone.0292922 (PMC10581466; doi:10.1371/journal.pone.0292922)
Supplement: S2 Table — (DOCX) [file pone.0292922.s002.docx]

**S2 Table: ICD 10 codes for Diagnosis used in this study.**

| **Diagnosis** | **ICD 10 Code** |
| --- | --- |
| **Cannabis Use Disorder** | F12.10 F12.120 F12.121 F12.122 F12.129 F12.13 F12.150 F12.151 F12.159 F12.180 F12.188 F12.19 F12.20 F12.220 F12.221 F12.222 F12.229 F12.23 F12.250 F12.251 F12.259 F12.280 F12.288 F12.29 F12.90 F12.920 F12.921 F12.922 F12.929 F12.93 F12.950 F12.951 F12.959 F12.980 F12.988 F12.99 |
| **Nicotine Use Disorder** | F17.200 F17.201 F17.203 F17.208  F17.209 F17.210 F17.211 F17.213 F17.218 F17.219 F17.220 F17.221 F17.223 F17.228 F17.229 F17.290 F17.291 F17.293 F17.298 F17.299 |
| **Stimulant Use Disorder** | F15.10 F15.11  F15.120 F15.121 F15.122 F15.129 F15.13 F15.14 F15.150 F15.151 F15.159 F15.180 F15.181 F15.182 F15.188 F15.19 F15.20 F15.21 F15.220 F15.221 F15.222 F15.229 F15.23 F15.24 F15.250 F15.251 F15.259 F15.280 F15.281 F15.282  F15.288 F15.29 F15.90 F15.91 F15.920 F15.921 F15.922 F15.929 F15.93  F15.94 F15.950 F15.951 F15.959 F15.980 F15.981  F15.982 F15.988 F15.99 |
| **Alcohol Use Disorder** | F10.10 F10.120 F10.121 F10.129 F10.130 F10.131 F10.132 F10.139 F10.14 F10.150 F10.151 F10.159 F10.180 F10.181 F10.182 F10.188 F10.19 F10.20 F10.220 F10.221 F10.229 F10.230 F10.231 F10.232 F10.239 F10.24 F10.250 F10.251 F10.259 F10.26 F10.27 F10.280 F10.281 F10.282 F10.288 F10.29 F10.920 F10.921 F10.929 F10.930 F10.931 F10.932 F10.939 F10.94 F10.950 F10.951 F10.959 F10.96 F10.97 F10.980 F10.981 F10.982 F10.988 F10.99 |
| **Cocaine use Disorder** | F14.10 F14.120 F14.121 F14.122 F14.129 F14.13 F14.14 F14.150 F14.151 F14.159 F14.180 F14.181 F14.18 F14.188 F14.19 F14.20 F14.220 F14.221 F14.222 F14.229 F14.23 F14.24 F14.250 F14.251 F14.259 F14.280 F14.281 F14.282 F14.288 F14.29 F14.90 F14.920 F14.921 F14.922 F14.929 F14.93 F14.94 F14.950 F14.951 F14.959 F14.980 F14.981 F14.982 F14.988 F14.99 |
| **ADHD** | F90.0 F90.1 F90.2 F90.8 F90.9 |
| **ODD** | F91.3 |
| **Conduct disorder** | F91.0 F91.1 F91.2 F91.8 F91.9 |
| **IDD** | F70 F71 F72 F73 F78.A1 F78.A9 F79 |
| **Suicide Attempt** | T14.91XA T14.91XD T1491.XS |
| **Self-harm** | X71.0XXA X71.0XXD X71.0XXS X71.1XXA X71.1XXD X71.1XXS X71.2XXA X71.2XXD X71.2XXS X71.3XXA X71.3XXD X71.8XXA X71.8XXD X71.8XXS X71.9XXA X71.9XXD X71.9XXS X72.XXXA X72.XXXD X72.XXXS X73.0XXA X73.0XXD X73.0XXS X73.1XXA X73.1XXD X73.1XXS X73.2XXA X73.2XXD X73.2XXS X73.8XXA X73.8XXD X73.8XXS X73.9XXA X73.9XXD X73.9XXS X74.01XA X74.01XD X74.01XS X74.02XA X74.02XD X74.02XS X74.09XA X74.09XD X74.09XS X74.8XXA X74.8XXD X74.8XXS X74.9XXA X74.9XXD X74.9XXS X75.XXXA X75.XXXD X75.XXXS X76.XXXA X76.XXXD X76.XXXS X77 X77.0XXA X77.0XXD X77.0XXS X77.1XXA X77.1XXD X77.1XXS X77.2XXA X77.2XXD X77.2XXS X77.3XXA X77.3XXD X77.3XXS X77.8XXA X77.8XXD X77.8XXS X77.9XXA X77.9XXD X77.9XXS X78.0XXA X78.0XXD X78.0XXS X78.1XXA X78.1XXD X78.1XXS X78.2XXA X78.2XXD X78.2XXS X78.8XXA X78.8XXD X78.8XXS X78.9XXA X78.9XXD X78.9XXS X79.XXXA X79.XXXD X79.XXXS X80.XXXA X80.XXXD X80.XXXS X81.0XXA X81.0XXD X81.0XXS X81.1XXA X81.1XXD X81.1XXS X81.8XXA X81.8XXD X81.8XXS X82.0XXA X82.0XXD X82.0XXS X82.1XXA X82.1XXD X82.1XXS X82.2XXA X82.2XXD X82.2XXS X82.8XXA X82.8XXD X82.8XXS X83.0XXA X83.0XXD X83.0XXS X83.1XXA X83.1XXD X83.1XXS X83.2XXA X83.2XXD X83.2XXS X83.8XXA X83.8XXD X83.8XXS |
| **Anxiety** | F41.0 F41.1 F41.3 F41.8 F41.9 |
| **Depression** | F33.0 F33.1 F33.2 F33.3 F33.8 F33.9 F32.0 F32.1 F32.2 F32.3 F32.89 F32.9 |
| **Eating disorder** | F50.00 F50.01 F50.02 F50.2 F50.81 F50.82 F50.89 F50.9 |
