# Supplementary material for: Correction: Cannabis use disorder, suicide attempts, and self-harm among adolescents: A national inpatient study across the United States
Source: PLoS One. 2023 Nov 30;18(11):e0294303. doi: 10.1371/journal.pone.0294303 (PMC10688704; doi:10.1371/journal.pone.0294303)
Supplement: S1 File — (PDF) [file pone.0294303.s001.pdf]

PLOS ONE

RESEARCH ARTICLE

Cannabis use disorder, suiscide attempts, and self-harm among adolescents: A national inpatient study across the United States

Adeolu Funso Oladunjoye[1], Elijah Li[2], Kammarauche Aneni[3‡*], Edore Onigu-Otite[1,2‡]

1 Menninger Department of Psychiatry, Baylor College of Medicine, Houston, Texas, United States of America, **2** School of Medicine, Baylor College of Medicine, Houston, Texas, United States of America, **3** Child Study Center, Yale School of Medicine, New Haven, Connecticut, United States of America

‡ KA and EO-O are Joint Senior Authors
* uche.aneni@yale.edu

Abstract

Background

Suicide is among the top three causes of adolescent mortality. There is a scarcity of research examining cannabis use and suicidal behavior in adolescents.

Objectives

To determine the association between cannabis use disorder (CUD) and suicide attempt/self-harm in a hospitalized sample of adolescents.

Methods

We conducted a cross-sectional observation study using data from the Nationwide Inpatient Sample collected over four years from January 1, 2016, through December 31, 2019. We included adolescents aged 10–19 hospitalized during the above period (N = 807,105). The primary outcome was suicide attempt/self-harm and the main predictor was CUD. The International Classification of Diseases Tenth Revision (ICD 10) diagnostic codes was used to identify a diagnosis of CUD, suicide attempt/self-harm, and other diagnoses included in the analyses. Adolescents diagnosed with CUD (n = 53,751) were compared to adolescents without CUD (n = 753,354). Univariate and multivariate logistic regressions were conducted to determine the association between CUD and suicide attempts/self-harm.

Results

807,105 adolescent hospitalizations were analyzed, of which 6.9% had CUD. Adolescents with CUD were more likely to be older (17 years vs. 15 years), female (52% vs. 48%), have depression (44% vs. 17%), anxiety (32% vs. 13%), an eating disorder (1.9% vs. 1.2%), ADHD (16.3% vs. 9.1%), Conduct Disorder (4.1% vs. 1.3%), Alcohol Use Disorder (11.9% vs. 0.8%), Nicotine Use Disorder (31.1% vs. 4.1%), Cocaine Use Disorder (5.4% vs. 0.2%), Stimulant Use Disorder (0.8% vs. 0.4%) and report suicide attempts/self-harm (2.8% vs.

OPEN ACCESS

Citation: Oladunjoye AF, Li E, Aneni K, Onigu-Otite E (2023) Cannabis use disorder, suiscide attempts, and self-harm among adolescents: A national inpatient study across the United States. PLoS ONE 18(10): e0292922. https://doi.org/10.1371/journal.pone.0292922

Editor: Rikinkumar S. Patel, Duke University Medical Center: Duke University Hospital, UNITED STATES

Received: July 1, 2023

Accepted: October 2, 2023

Published: October 17, 2023

Copyright: © 2023 Oladunjoye et al. This is an open access article distributed under the terms of the Creative Commons Attribution License, which permits unrestricted use, distribution, and reproduction in any medium, provided the original author and source are credited.

Data Availability Statement: The data used in this study is available publicly at https://hcup-us.ahrq.gov/db/nation/nis/nisdde.jsp

Funding: KA is supported by National Institute on Drug Abuse (NIDA) Diversity Supplement award #3UH3DA050251-03S1, Doris Duke Charitable Foundation award #20220145, and the Yale Center for Clinical Investigation CTSA Grant award #UL1 TR001863. Its contents are solely the responsibility of the authors and do not necessarily represent the

official view of NIH. The funders had no role in study design, data collection and analysis, decision to publish, or preparation of the manuscript.

Competing interests: The authors have declared that no competing interests exist.

0.9%) [all ps<0.001]. After adjusting for potential confounders, CUD was associated with a higher risk of suicide attempts/self-harm (OR = 1.4, 95% CI 1.3–1.6, p <0.001).

Post-hoc analyses showed the presence of depression moderated the association between CUD and suicide attempts/self-harm in that adolescents with CUD and depression had 2.4 times the odds of suicide attempt/self-harm compared to those with CUD but no depression after controlling for potential confounders (p<0.001).

Conclusions

Our study provides evidence for the association between CUD and suicide risk among hospitalized adolescents and underscores the importance of recognizing and addressing co-occurring mental and substance use disorders along with CUD to mitigate suicide risk. Identifying high-risk adolescents in inpatient settings provides an opportunity for intervention.

Introduction

Suicide is one of the top three leading causes of adolescent death, accounting for about 1–2 in 10 deaths among 12–19-year-olds [1,2]. The history of a suicide attempt is one of the strongest predictors of completed suicide in adolescents [3,4]. The prevalence of suicide, suicide planning, suicide attempts, and suicide-related hospitalizations are rising among adolescents [5,6]. These concerning statistics underscore the importance of identifying risk factors for suicidal behaviors to develop and implement effective solutions.

Substance use is associated with an increased risk of engaging in suicidal behaviors [7,8]. Cannabis is the most used illegal substance worldwide and in the United States. In 2019, 18% of Americans reported using cannabis at least once [9]. In 2019, 37% of US high school students reported lifetime use of marijuana, and 22% reported use in the past 30 days [10]. Almost one in five adolescents had used cannabis frequently between ages 13 and 17 (26.6% of males, 9.8% of females) [11]. The legalization of cannabis in some states has contributed to a lower risk-perception including among youth [12]. Coupled with increasing availability, the rise in cannabis use among adolescents and young adults is concerning in light of evidence that adolescents are susceptible to its harmful effects on brain development and neurocognitive functioning [13,14]. In some studies, evidence suggests that at this stage of development, the brain is exposed to effects that directly impact behavioral changes, including suicidal behavior [15–17].

Adolescence is a critical period for neurocognitive, emotional, psychological, and social development, with the various processes involved extending into early adulthood [18]. Animal studies demonstrate that cannabis exposure in adolescence alters gene expression associated with the structural maturation of neuronal cells in the prefrontal cortex and with reward and stress reactivity in the amygdala [19,20]. Alteration in the endocannabinoid system with early cannabis use can result in persisting neurological effects leading to poor cognitive and emotional outcomes lasting into adulthood [21]. Human studies suggest that at this stage of neurodevelopment, exposure to the effects of exogenous cannabinoids directly impacts behavioral changes, including suicidal behavior [15–17]. Recent evidence suggests the activation of the CB1 receptor by exogenous cannabinoids can diminish the production of neuronal growth factor and affect other signaling cascades involved in synapsis formation [16]. Some studies have found that cannabis exposure during adolescence is associated with altered cerebral cortical development increasing the risk of neurocognitive problems such as memory, learning,

persisting attentional deficits, and impulse control problems. Such changes in neurocognitive function due to cannabis use may increase the risk of occurrence or worsening of other mental health symptoms [13]. This in turn, may increase the risk of maladaptive behaviors, which prevent individuals from adapting to the changes in various aspects of life that come with adolescence [16]. Such challenges can increase the risk of stress, depression, and anxiety, increasing the risk of suicidal behaviors [15].

Most studies that examined the association between substance use and suicidal behavior in adolescents have not focused on cannabis, and available studies have been predominantly conducted in the adult population [15,22–25]. Existing studies examining the association between cannabis use and suicidal behaviors among adolescents have focused on the general population [26,27] or included a small inpatient population limited to adolescents admitted for psychiatric hospitalization [28,29]. Adolescents, including those with ongoing cannabis use, most often present for care in medical settings for medical problems and may not be seen by a psychiatrist in these settings unless a consult is requested and a provider is available, an increasing challenge in context of mental health provider shortage. Individuals with cannabis use are often hospitalized for medical reasons, including some due to complications of cannabis use [30–32]. Additionally, due to a national shortage of psychiatric beds, adolescents with mental health problems including CUD are increasingly hospitalized on non-psychiatric pediatric beds [33]. Therefore, this study builds on existing studies using a nationally representative adolescent inpatient that includes both a psychiatric and non-psychiatric sample spanning four years and aims to examine the association between Cannabis use disorder (CUD) and suicidal attempt/self-harming behavior among adolescent hospitalizations.

We hypothesized that among hospitalized adolescents, adolescents with CUD would have a higher likelihood of suicide attempts/self-harm behaviors compared to adolescents without CUD. Understanding the association between cannabis use disorder and suicidal behaviors among adolescents in the inpatient setting will expand our understanding of substance use risk factors for suicidal behaviors among hospitalized adolescents and inform targeted interventions that can be implemented in the inpatient setting.

Materials and methods

Study design and data sources

A cross-sectional study was conducted based on the Nationwide Inpatient Sample (NIS) data administered by the Agency for Healthcare Research and Quality, a part of the Healthcare Cost and Utilization Project (HCUP) [34]. The NIS is the largest publicly available all-payer inpatient care database designed to produce U.S. regional and national estimates of inpatient utilization, access, cost, quality, and outcomes. The study period began in 2016, the first full year the International Classification of Diseases Tenth Revision Clinical Modification (ICD-10-CM) billing codes were available in the NIS database up until 2019. All adolescent (10–19 years) admissions from January 1, 2016, through December 31, 2019, were included in the analyses using the World Health Organization age definition of adolescents defined as the phase of life between child and adulthood from ages 10 to 19. Since the database is de-identified and publicly available, ethical clearance or Institutional Review Board approval was not required. Also, authors had no access to any information that could identify individual participants during or after data collection.

Study population and characterization of variables

All adolescent inpatient hospitalizations (ages 10–19) from the NIS database between 2016 and 2019 were included totaling about 807,105. Within this sample, we compared adolescents

admitted with an ICD-10 diagnosis of CUD to adolescents without, regardless of whether CUD was the primary or comorbid diagnosis. Similarly, we compared adolescents admitted with ICD-10 codes of suicide attempt/self-harm to those without. We also identified other co-occurring conditions in these adolescent age groups, including diagnosis of depression, anxiety, eating disorder, Attention Deficit Hyperactivity Disorder (ADHD), Intellectual and Developmental Disabilities (IDD), Conduct disorder, Substance Use Disorders (Alcohol, Nicotine, Cocaine and Stimulants).

Patient demographics and comorbidity characteristics

Patient-level characteristics from the database included age (10–19 years), race (white, Black, Hispanic, Asian, and others), primary payer (public, private, and self-pay), regions of the US (northeast, south, mid-west/north central, and west), the All Patients Refined Diagnosis Related Groups (APRDRG) severity of illness score (minor, moderate, major and extreme) and discharge disposition (home, short term facility, skilled nursing home, home health care, discharge against medical advice (AMA) and died). Co-occurring conditions included depression, anxiety, and eating disorder, ADHD, IDD, Conduct disorder, Substance Use Disorders (Alcohol, Nicotine, Cocaine and Stimulants).

Statistical analysis

The prevalence of cannabis use disorder and suicide attempt/self-harm were determined in this adolescent population from 2016–2019. Demographic and clinical characteristics of hospitalizations with CUD were compared to hospitalizations without CUD. Analyses used only non-missing data, constituting less than 10% of the sample. We conducted univariate and multivariate logistic regression analyses to determine the association between CUD and suicide attempt/self-harm. In addition to this first model that combined suicide attempt/self-harm into a single outcome, we conducted separate analyses with suicide attempt and self-harm as two separate outcomes to determine if the association with CUD differed by each outcome. Multivariable logistic regression analyses were adjusted for independent factors, including age, sex, race, primary payer, the severity of illness score, and illness comorbidities, including depression, anxiety, eating disorder, ADHD, IDD, Conduct disorder, Substance Use Disorders (Alcohol, Nicotine, Cocaine and Stimulants). Post-hoc analyses was conducted to explore the moderating effect of depression and anxiety on the association between CUD and suicide attempt/self-harm. We focused on depression and anxiety as these are the two most common mental disorders in adolescence. STATA version 15.0 (College Station, TX) was used for all statistical analyses. A P-value of <0.05 and a 95% confidence interval (CI) was used in the analysis.

Results

Table 1 describes the demographic and clinical characteristics of adolescents in the total sample and in adolescents with and without CUD. The prevalence of CUD in this population was found to be about 6.9%. The overall mean age was 16 years. Adolescents in the CUD group were more likely to be older (17 years vs. 15 years, $p<0.001$), more likely to be females (52% vs. 48%, $p<0.001$), more likely to be diagnosed with depression (44% vs. 17%, $p<0.001$), anxiety (32% vs. 13%, $p<0.001$), an eating disorder (1.9% vs. 1.2%, $p<0.001$), ADHD (16.3% vs. 9.1%, $p<0.001$), Conduct Disorder (4.1% vs. 1.3%), and suicide attempts (2.8% vs. 0.9%, $p<0.001$). Adolescents with CUD were also more likely to have Alcohol Use Disorder (11.9% vs. 0.8%, $p<0.001$), Nicotine Use Disorder (31.1% vs. 4.1%, $p<0.001$), Cocaine Use Disorder (5.4% vs. 0.2%, $p<0.001$) and Stimulant Use Disorder (0.8% vs. 0.4%, $p<0.001$). Over half of the

Table 1. Patient demographics and clinical characteristics of adolescent hospitalizations with cannabis use disorder.

Variable name	All (n = 807,105)	Study Groups		
		Non- CUD group (n = 753,354)	CUD group (n = 53,751)	*P Value*
Mean Age (±SE)	15.82 ± 0.00	15.73± 0.00	17.20 ± 0.01	<0.0001
Age category				
10–14	30.7	32.3	8.0	
15–19	69.3	67.7	92.0	<0.0001
Sex				
Female	62.2	62.9	51.6	
Male	37.8	37.1	48.4	<0.0001
Race, %				
White	51.0	50.6	56.9	
Black	19.2	19.1	20.4	
Hispanic	21.5	22.0	15.2	
Asian	2.5	2.5	1.5	
Others	5.8	5.8	6.0	<0.0001
Insurance, %				
Public	55.0	55.1	53.5	
Private	41.4	41.4	40.8	
Self- Pay	3.6	3.5	5.7	<0.0001
Region, %				
Northeast	16.1	16.0	17.4	
Mid-West/North Central	23.4	23.0	30.0	
South	40.1	40.5	33.6	
West	20.4	20.5	19.0	<0.0001
APRDG severity				
Minor	40.5	40.8	35.6	
Moderate	41.8	41.2	51.2	
Major	14.0	14.2	11.0	
Extreme	3.7	3.8	2.2	<0.0001
CUD as primary diagnosis	0.1	0.0	2.1	<0.0001
Co-morbidities				
Depression	18.7	16.9	43.9	<0.0001
Anxiety	14.5	13.3	31.8	<0.0001
Eating Disorder	1.2	1.2	1.9	<0.0001
ADHD	9.6	9.1	16.3	<0.0001
Conduct disorder	1.5	1.3	4.1	<0.0001
IDD	1.3	1.3	0.4	<0.0001
Other Substance use disorders				
Alcohol use disorder	1.5	0.8	11.9	<0.0001
Nicotine use disorder	5.9	4.1	31.1	<0.0001
Cocaine use disorder	0.5	0.2	5.4	<0.0001
Stimulant use disorder	0.8	0.4	0.8	<0.0001
Suicide Attempt/Self-harm				
Yes	1.0	0.9	2.8	
No	99.0	99.1	97.2	<0.0001
Disposition on discharge				
Home	91.8	92.0	88.7	

(*Continued*)

Table 1. (Continued)

Variable name	All (n = 807,105)	Study Groups		
		Non- CUD group (n = 753,354)	CUD group (n = 53,751)	*P Value*
**Short term facility**	1.3	1.3	1.3	
**Skilled nursing home**	3.7	3.4	7.1	
**Home health care**	2.2	2.4	0.9	
**Discharge AMA**	0.7	0.6	1.9	
**Died**	0.3	0.3	0.1	<0.0001

n: Sample number; SE: Standard error; %: Percentage; CUD–Cannabis use disorder.

https://doi.org/10.1371/journal.pone.0292922.t001

adolescents in the CUD group were white (57%) and had a moderate APRDRG severity (51%). Insurance coverage varied, with public insurance (55.0%) being the predominant insurance coverage, followed by private insurance (41.4%) and self-pay 3.6%. Most patients were from the southern part of the country, with an overall proportion of about 40.1% ($p<0.001$). We dropped 76,646 participants from the dataset with missing values among the variables of interest, given that the proportion of missing data was less than 10% and, therefore, unlikely to impact the internal validity of our findings. Missing data included: sex (n = 133), race (n = 44,821), insurance (n = 32,848), APRDRG status (n = 975), and disposition on discharge (n = 603).

Factors associated with CUD and suicide attempt/self-harm in adolescent hospitalizations. In univariate logistic regression, having a CUD diagnosis increased the odds of having a diagnosis of suicide attempt/self-harm by 218%. In a multivariate logistic regression model after controlling for age, sex, race, insurance, APRDRG severity, and other co-occurring disorders (Depression, Anxiety, Eating Disorder, ADHD, IDD, Conduct Disorder, Alcohol Use Disorder, Nicotine Use Disorder, Cocaine Use Disorder, Stimulant Use Disorder), adolescents with a diagnosis of CUD had 40% higher odds of being engaged in suicide attempt/self-harm (95% CI: 1.32–1.55, $p<0.001$) (Table 2). As noted above, we also conducted analysis with suicide attempts and self-harm as separate outcomes (See S1 Table). In the multivariate analysis, CUD was associated with 40% higher odds of suicide attempts and 40% higher odds of self-harm (S1 Table).

We found that compared to adolescents with no CUD and no depression, after controlling for potential confounders, higher odds of suicide attempt/self-harm occurred in adolescents who had no CUD with depression (OR:8.6, 95% CI: 7.9, 9.4), CUD with no depression (3.8, 95% CI: 3.4, 4.4) and CUD with depression (OR: 9.0, 95% CI: 8.0, 10.2) (Table 3). Likewise, compared to adolescents with no CUD and no anxiety, after controlling for potential confounders, higher odds of suicide attempts/self-harm was found in adolescents with no CUD and anxiety (OR: 1.5, 95% CI: 1.4, 1.6), CUD with no anxiety (OR: 1.8, 95% CI: 1.6, 2.0), CUD with anxiety (OR: 1.6, 95% CI: 1.4, 1.8) (Table 3).

When we examined the moderating effect of depression on the association between CUD and suicide risk, we found that the presence of depression moderated this association such that those with CUD and depression had 2.4 times the odds of suicide risk compared to those with CUD but no depression after controlling for potential confounders ($p<0.001$) (Fig 1). Likewise, adolescents with CUD and anxiety had 1.6 times the odds of suicide risk compared to adolescents with CUD but no anxiety ($p<0.001$) (Fig 1). However, this association was lost after controlling for potential confounders ($p>0.05$) (Fig 1).

Table 2. Factors associated with Cannabis use disorder and suicide attempt/self-harm in adolescent hospitalizations.

Variable name	Univariate analysis (Unadjusted OR)	Multivariate analysis (Adjusted OR)
**Mean Age (±SE)**	1.00 (0.99–1.01)	0.98 (0.97–0.99) ***
**Age category**		
**10–14**	Reference	
**15–19**	1.05 (0.99–1.12)	0.89 (0.84–0.95) ***
**Cannabis use disorder**		
**No**	Reference	Reference
**Yes**	3.18 (2.97–3.41) ***	1.43 (1.32–1.55) ***
**Sex**		
**Male**	Reference	Reference
**Female**	1.29 (1.22–1.36) ***	1.18 (1.11–1.25) **
**Race, %**		
**White**	Reference	Reference
**Black**	0.49 (0.46–0.54) ***	0.71 (0.65–0.77) ***
**Hispanic**	0.50 (0.45–0.57) ***	0.78 (0.69–0.87) ***
**Asian**	0.76 (0.63–0.91) **	1.04 (0.87–1.25)
**Others**	0.80 (0.71–0.91) **	0.95 (0.84–1.07)
**Insurance, %**		
**Public**	Reference	Reference
**Private**	1.30 (1.21–1.39) ***	1.02 (0.95–1.10)
**Self- Pay**	1.50 (1.31–1.74) ***	1.40 (1.22–1.61) ***
**APRDG severity**		
**Minor**	Reference	Reference
**Moderate**	1.45 (1.37–1.54) ***	1.07 (1.01–1.14) **
**Major**	0.94 (0.86–1.03)	0.88 (0.80–0.97) *
**Extreme**	1.17 (0.01–1.34) *	1.45 (1.26–1.68) ***
**Co-morbidities**		
**Depression**	9.55 (8.91–10.22) ***	7.06 (6.52–7.66) ***
**Anxiety**	4.09 (3.82–4.37) ***	1.32 (1.22–1.42) ***
**Eating Disorder**	3.78 (3.36–4.25) ***	1.45 (1.29–1.64) ***
**ADHD**	2.32 (2.18–2.47) ***	1.41 (1.32–1.50) ***
**IDD**	0.92 (0.74–1.14) **	1.40 (1.12–1.76) **
**Conduct disorder**	2.16 (1.88–2.48) **	1.32 (1.12–1.55) **
**Substance use disorders**		
**Alcohol use disorder**	3.96 (3.57–4.40) ***	1.53 (1.36–1.72) ***
**Nicotine Use disorder**	2.81 (2.60–3.03) ***	1.48 (1.36–1.62) ***
**Cocaine Use disorder**	3.07 (2.55–3.70) ***	1.14 (0.93–1.40)
**Stimulant Use disorder**	3.01 (2.60–3.51) ***	1.27 (1.08–1.50) ***

SE: Standard error, %: Percentage; Ref–reference group

*<0.05

**<0.01

***<0.001.

https://doi.org/10.1371/journal.pone.0292922.t002

Discussion

We conducted a study to determine the association between cannabis use disorder (CUD) and suicidal attempt/self-harm using a nationally representative inpatient adolescent sample. The

Table 3. Cannabis use disorder, depression, anxiety, and suicide attempt/self-harm in adolescent hospitalizations.

Study Group	Variable name	Univariate analysis (Unadjusted OR)	Multivariate analysis (Adjusted OR)
CUD*Depression	No CUD + No Depression	reference	reference
	NO CUD + Depression	10.81 (10.07–11.60) ***	8.62 (7.93–9.38) ***
	CUD + No Depression	4.72 (4.23–5.27) ***	3.83 (3.38–4.35) ***
	CUD + Depression	13.04 (11.80–14.41) ***	9.04 (8.02–10.20) ***
CUD*Anxiety	No CUD + No Anxiety	reference	reference
	NO CUD + Anxiety	4.43 (4.13–4.76) ***	1.46 (1.34–1.59) ***
	CUD + No Anxiety	3.85 (3.53–4.19) ***	1.79 (1.62–1.98) ***
	CUD + Anxiety	6.25 (5.62–6.96) ***	1.62 (1.43–1.82) ***

SE: Standard error, %: Percentage; Ref–reference group;
*<0.05,
**<0.01,
***<0.001.

https://doi.org/10.1371/journal.pone.0292922.t003

present study found that CUD was strongly associated with suicidal attempts/self-harm both as a combined outcome and separate outcomes (suicide attempt and self-harm) after controlling for potential confounders. This finding is consistent with previous non-inpatient epidemiological studies [35,36], and a prior adolescent inpatient psychiatric sample [29], but in contrast to the adolescent inpatient study by Sellers et al., which found no independent association between cannabis use and self-harm [28], possibly due to the small sample size.

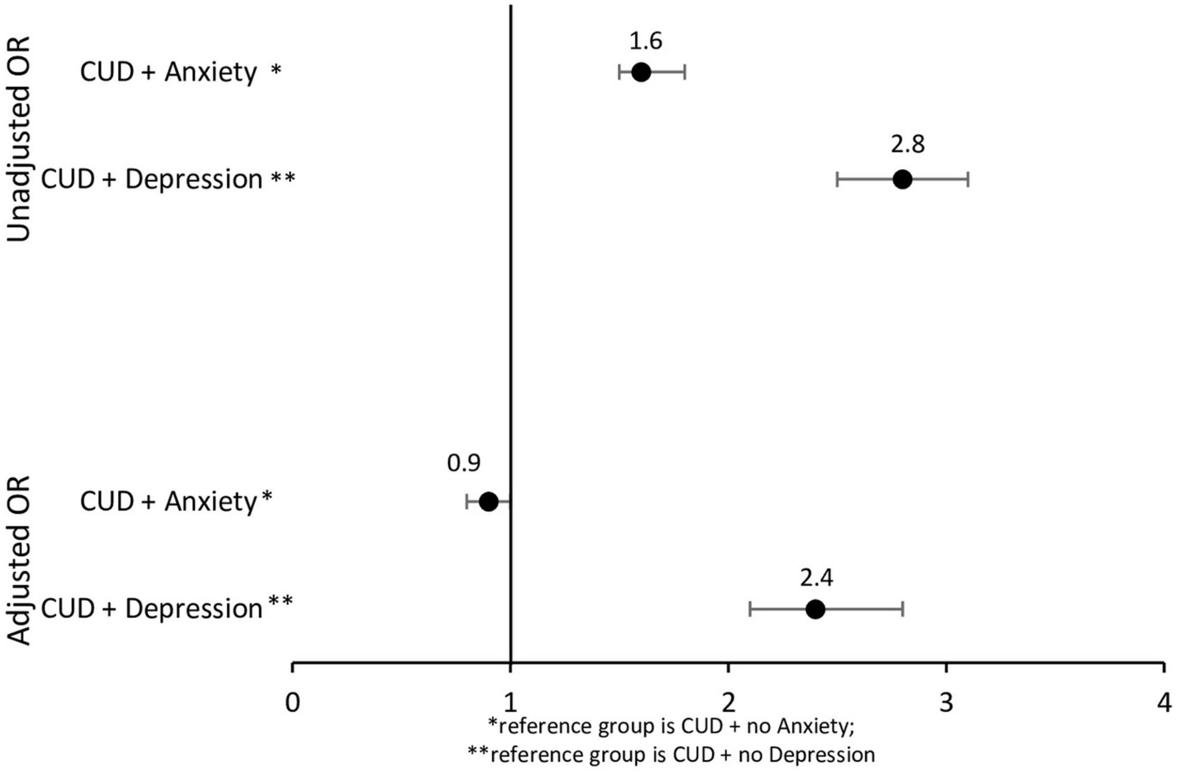

Fig 1. Moderation analyses: Odds of suicide attempt/self-harm among adolescents with CUD and depression or anxiety compared to Adolescents with CUD but no depression or anxiety.

https://doi.org/10.1371/journal.pone.0292922.g001

Findings from our study suggest an independent association between CUD and suicidal behaviors and non-suicidal self-harm and underscore the importance of addressing CUD among adolescents in inpatient settings. Clinically, while non-accidental self-harm may be clear, the degree of suicidal intent behind such actions can be more obscure. Since our study is cross-sectional, we cannot determine whether CUD preceded suicidal behaviors or vice-versa. Nevertheless, the inpatient setting provides a unique opportunity to engage adolescents in addressing cannabis use regardless of their reason for hospitalization, particularly those adolescents who may not otherwise have been involved in treatment prior to the hospitalization. The inpatient setting may also lend itself to automated, evidence-based digital interventions that the adolescent can access without the need for additional provider manpower. However, potential limitations in implementing these interventions can arise from limited or no access to digital devices while on the inpatient unit, the severity of illness that may hinder the adolescent ability to engage in self-guided digital interventions. However, the inpatient setting can allow for the identification of high-risk adolescents who require follow-up substance use treatment. The inpatient setting also provides an opportunity to implement multidisciplinary approaches that leverage healthcare providers, families, and community resources to develop a comprehensive plan to support the adolescent. Future studies are needed to investigate inpatient approaches to inform specific intervention strategies.

Our study also found that depression, anxiety, eating disorders, ADHD, Conduct Disorder, Alcohol Use Disorder, Nicotine Use Disorder, Cocaine Use Disorder and Stimulant Use Disorder were significantly associated with CUD. In addition, except for Cocaine Use Disorder, these factors were also strongly associated with suicidal attempts/self-harm independent of CUD. Furthermore, in the analyses that examined suicide attempts and self-harm as separate outcomes, depression, anxiety, ADHD, and stimulant use disorder were associated with suicide attempts and self-harm independent of CUD. Although this is a cross-sectional study which cannot establish causation, other studies have shown that cannabis use increases the risk for developing other illicit drug use (the gateway hypothesis) [37] as well as other mental disorders during adolescence [38,39], specifically depression and anxiety, which can then further increase the risk of self-harm or suicide [15]. Our study revealed that hospitalized adolescents with CUD had over double the rates of depression, anxiety, ADHD, Conduct Disorder, and all the substance use disorders included (Alcohol, Nicotine, Cocaine and Stimulant) when compared to adolescents without CUD. We also found that comorbid CUD and depression were associated with higher odds of suicidal behavior compared to CUD alone, similar to findings from a recent publication [27]. Both depression and anxiety, being internalizing disorders, are often chronic and less readily detected. A prospective cohort study lasting seven years found that frequent cannabis use in teenage girls predicted later depression and anxiety, with daily users carrying the highest risk [40]. Similarly, a systematic review and meta-analysis found that that cannabis use in adolescence increased the risk of developing depression and anxiety in young adulthood [41]. These findings highlight the critical importance of a thorough mental health and substance use history when assessing adolescents in the inpatient setting and at all patient encounters, including in primary care and emergency room settings. In addition, our findings underscore the importance of the dual diagnosis approach of addressing both substance use and co-occurring mental disorders to alleviate suicide risk among adolescents [42,43].

In contrast to a prior nationally representative study among non-inpatient adolescents which showed no racial/ethnic differences in the association between cannabis use and suicidal risk [44], our study also revealed lower odds of suicidal/self-harm behaviors among Black and Hispanic adolescents compared to white adolescents. These lower odds appeared to be in relation to the risk for self-harm and not suicide attempts (See S1 Table) in contrast to more recent

studies showing higher rates of suicide attempts among Black adolescents compared to the white adolescents [45,46]. Although, historical data shows comparatively lower rates of suicide among Black and Hispanic adolescents, our finding of no difference in the risk for suicide attempts between Black and white adolescents, would be consistent with the alarming rise in suicide rates among Black adolescents [47]. Regarding self-harm, studies investigating racial/ethnic differences in non-suicidal self-harm have revealed mixed findings [48]. However, a recent systematic review found higher prevalence among non-Hispanic white adolescents and specifically Black boys [48]. Future studies should further clarify these findings, investigate potential racial and sex differences and investigate potential reasons for these differences.

Additional outcomes of this study suggest that female sex, being from the US Mid-west/North Central or South, having a self-pay payment option, and having co-occurring psychiatric disorders are associated with CUD and suicide attempt/self-harm among adolescents' hospitalizations. It is plausible that having a self-pay payment option may reflect a higher socioeconomic status and the ability to seek and obtain healthcare. Other studies are unclear about the relationships between cannabis use, CUD and suicidal attempts or self-harm, and other variables such as sex, insurance type, and location [49,50]. Although the relationship remains poorly understood, consistent with our study, other studies find that females are more susceptible to adverse outcomes after cannabis use [42,51,52]. However, some studies report that men consume more cannabis than by women and this consumption is associated with co-occurring suicide behaviors [53,54]. When we separated out the outcomes, female sex was associated with self-harm but not suicide attempts. This difference may relate to findings indicating that females are more likely to report non-suicide self-injury behaviors than men [55]. Future studies are needed to investigate these findings.

Strengths and limitations

The present study contributes to the literature on a subject that needs to be vitally addressed by looking at a large sample size of nationally representative hospitalized adolescents. Including adolescents with CUD in non-psychiatric beds is a unique feature of this study since many adolescents with CUD are admitted for non-psychiatric reasons and/or admitted to non-psychiatric beds. The findings of the present study should be interpreted in the context of possible limitations. As with any cross-sectional study, our study cannot establish any causative effects between cannabis use disorder, suicidal behaviors, depression, or anxiety. While the present study elucidates various associations between CUD and suicide and self-harm in adolescent hospitalizations, no causal conclusions can be drawn. Since this study included both psychiatric and non-psychiatric hospitalizations, we cannot determine if our findings are applicable to adolescents admitted for psychiatric hospitalizations versus not which might have implications for developing and administering interventions to mitigate suicide risk in the inpatient setting. In addition, our findings are not generalizable to non-inpatient samples. Given that diagnosis may be inconsistently reported and document, CUD may be underdiagnosed in the current sample which would result in under-estimation of associations.

The lack of assessment of severity of CUD is a limitation as it may be reasonable to think that adolescents with heavier cannabis use may experience more mental health problems resulting in increased rates of self-harm or suicide. Those with more severe CUD may also have higher rates of depression and anxiety. Although it was not the primary focus of this study, the lack of assessment of the severity of depression is a limitation given that adolescents with more severe depression may likely have more suicide attempts or self-harm. The measurement of suicidality in adolescents also constitutes a limitation. The intent behind suicidal behaviors is often unclear, sometimes even to the adolescents involved and it is sometimes

difficult to confirm if or when self-injury was intended for death, leaving the intent undetermined [56,57]. As much as we intended to include those with intentional suicidal self-injury, it is possible that some of our study population included those who experienced non- suicidal self-injury (NSSI). However, what is clear is that NSSI is associated with a higher risk of suicide attempts and suicide [58–60]. Regardless of these limitations, our findings underscore the importance of assessing suicide risk in patients admitted with CUD and highlight the need to undertake a dual diagnosis approach to mitigate suicide risk among adolescents.

Conclusion

To our knowledge, this is the first study examining the association between cannabis use and suicidal attempts or self-harm in a nationally representative adolescent hospital sample. The results of this study indicate that suicide attempts and self-harm are associated with CUD in hospitalized adolescents and that this association is moderated by depression. Identifying high-risk adolescents during hospitalizations, regardless of their reason for admission, and initiating early interventions at this point of care with subsequent follow-up care and support should be a key component of comprehensive suicide prevention strategies in this vulnerable population.

Our study revealed high levels of co-occurring depression, anxiety, ADHD, Alcohol Use Disorder and Nicotine Use Disorder in adolescents with CUD. Depression and anxiety, both being internalizing disorders, may be poorly recognized or undertreated leaving adolescents dealing with their symptoms in unhealthy ways, including by using cannabis, further worsening their condition. Most adolescents, with mental health and substance use problems including those with ongoing cannabis use, do not present to psychiatrists, who, as a specialty, are in very short supply. Rather, most adolescents with ongoing cannabis use most often present to non-psychiatric visits such as to annual physical, visits with primary care, or emergency care. Early intervention including identification and optimal treatment of comorbid mental and substance use disorders could result in reduction of symptoms, potentially reduce the risk of progression to self-harming behavior. In adolescents with CUD, the dual diagnosis approach may result in more robust patient outcomes.

With the recent rise in Cannabis use among adolescents, and the increased risk of mental health problems including self-harm/suicide, findings from this study underscore the need for targeted prevention efforts that address cannabis use among adolescents in inpatient settings. Our study highlights the need for policies that promote adolescent mental health education and limit access to cannabis use during adolescence which remains a vulnerable neurodevelopment stage.

Supporting information

S1 Table. Multivariate analysis testing association with Cannabis use disorder (CUD) and suicide attempt / CUD & self-harm in adolescent hospitalizations.
(DOCX)

S2 Table. ICD 10 codes for diagnosis used in this study.
(DOCX)

Acknowledgments

The authors would like to thank Dr. Bunmi Oladunjoye who provided consultation on statistical analysis. Appreciation also goes to the Agency for Healthcare Research and Quality who

developed this database under the health cost and utilization project through a federal-states-industry partnership.

Author Contributions

Conceptualization: Adeolu Funso Oladunjoye, Kammarauche Aneni, Edore Onigu-Otite.

Formal analysis: Adeolu Funso Oladunjoye, Kammarauche Aneni.

Investigation: Adeolu Funso Oladunjoye, Kammarauche Aneni, Edore Onigu-Otite.

Methodology: Adeolu Funso Oladunjoye, Kammarauche Aneni, Edore Onigu-Otite.

Supervision: Kammarauche Aneni, Edore Onigu-Otite.

Visualization: Adeolu Funso Oladunjoye, Kammarauche Aneni, Edore Onigu-Otite.

Writing – original draft: Adeolu Funso Oladunjoye, Elijah Li, Edore Onigu-Otite.

Writing – review & editing: Adeolu Funso Oladunjoye, Elijah Li, Kammarauche Aneni, Edore Onigu-Otite.

References

1. Kann L, McManus T, Harris WA, Shanklin SL, Flint KH, Queen B, et al. Youth Risk Behavior Surveillance - United States, 2017. MMWR Surveill Summ. 2018; 67: 1–114. https://doi.org/10.15585/mmwr.ss6708a1 PMID: 29902162
2. Minino AM. Mortality Among Teenagers Aged 12–19 Years: United States, 1999–2006. PsycEXTRA Dataset. American Psychological Association (APA); 2010. https://doi.org/10.1037/e665432010-001
3. Cavanagh JTO, Carson AJ, Sharpe M, Lawrie SM. Psychological autopsy studies of suicide: a systematic review. Psychological Medicine. 2003; 33: 395–405. https://doi.org/10.1017/s0033291702006943 PMID: 12701661
4. Hawton K, Arensman E, Wasserman D, Hultén A, Bille-Brahe U, Bjerke T, et al. Relation between attempted suicide and suicide rates among young people in Europe. J Epidemiol Community Health. 1998; 52: 191–194. https://doi.org/10.1136/jech.52.3.191 PMID: 9616425
5. Curtin, Sally C. State suicide rates among adolescents and young adults aged 10–24: United States, 2000–2018. National Vital Statistics Report. 2020;69. Available: https://stacks.cdc.gov/view/cdc/93667.
6. Plemmons G, Hall M, Doupnik S, Gay J, Brown C, Browning W, et al. Hospitalization for Suicide Ideation or Attempt: 2008–2015. Pediatrics. 2018; 141. https://doi.org/10.1542/peds.2017-2426 PMID: 29769243
7. Kokkevi A, Richardson C, Olszewski D, Matias J, Monshouwer K, Bjarnason T. Multiple substance use and self-reported suicide attempts by adolescents in 16 European countries. European Child & Adolescent Psychiatry. 2012; 21: 443–450. https://doi.org/10.1007/s00787-012-0276-7 PMID: 22535305
8. Kokkevi A, Rotsika V, Arapaki A, Richardson C. Adolescents' self-reported suicide attempts, self-harm thoughts and their correlates across 17 European countries. Journal of Child Psychology and Psychiatry. 2011; 53: 381–389. https://doi.org/10.1111/j.1469-7610.2011.02457.x PMID: 21895649
9. Substance Abuse and Mental Health Services Administration. Key Substance Use and Mental Health Indicators in the United States: Results from the 2019 National Survey on Drug Use and Health (HHS Publication No. PEP20-07-01-001, NSDUH Series H-55). HHS. 2019; 114.
10. Jones CM. Prescription Opioid Misuse and Use of Alcohol and Other Substances Among High School Students — Youth Risk Behavior Survey, United States, 2019. MMWR Suppl. 2020; 69. https://doi.org/10.15585/mmwr.su6901a5 PMID: 32817608
11. Shanahan L, Steinhoff A, Bechtiger L, Copeland WE, Ribeaud D, Eisner M, et al. Frequent teenage cannabis use: Prevalence across adolescence and associations with young adult psychopathology and functional well-being in an urban cohort. Drug and Alcohol Dependence. 2021; 228: 109063. https://doi.org/10.1016/j.drugalcdep.2021.109063 PMID: 34601277
12. Mennis J, McKeon TP, Stahler GJ. Recreational cannabis legalization alters associations among cannabis use, perception of risk, and cannabis use disorder treatment for adolescents and young adults. Addictive Behaviors. 2023; 138: 107552. https://doi.org/10.1016/j.addbeh.2022.107552 PMID: 36413909

13. Schweinsburg AD, Brown SA, Tapert SF. The influence of marijuana use on neurocognitive functioning in adolescents. Curr Drug Abuse Rev. 2008; 1: 99–111. https://doi.org/10.2174/1874473710801010099 PMID: 19630709

14. Volkow ND, Swanson JM, Evins AE, DeLisi LE, Meier MH, Gonzalez R, et al. Effects of Cannabis Use on Human Behavior, Including Cognition, Motivation, and Psychosis: A Review. JAMA Psychiatry. 2016; 73: 292–297. https://doi.org/10.1001/jamapsychiatry.2015.3278 PMID: 26842658

15. Borges G, Benjet C, Orozco R, Medina-Mora M-E, Menendez D. Alcohol, cannabis and other drugs and subsequent suicide ideation and attempt among young Mexicans. Journal of Psychiatric Research. 2017; 91: 74–82. https://doi.org/10.1016/j.jpsychires.2017.02.025 PMID: 28325681

16. Dhein S. Different Effects of Cannabis Abuse on Adolescent and Adult Brain. Pharmacology. 2020; 105: 609–617. https://doi.org/10.1159/000509377 PMID: 32629444

17. Meruelo AD, Castro N, Cota CI, Tapert SF. Cannabis and alcohol use, and the developing brain. Behav Brain Res. 2017/02/20 ed. 2017; 325: 44–50. https://doi.org/10.1016/j.bbr.2017.02.025 PMID: 28223098

18. Toga AW, Thompson PM, Sowell ER. Mapping Brain Maturation. FOCUS. 2006; 4: 378–390. https://doi.org/10.1016/j.tins.2006.01.007 PMID: 16472876

19. Miller ML, Chadwick B, Dickstein DL, Purushothaman I, Egervari G, Rahman T, et al. Adolescent exposure to Δ(9)-tetrahydrocannabinol alters the transcriptional trajectory and dendritic architecture of prefrontal pyramidal neurons. Mol Psychiatry. 2018/10/03 ed. 2019; 24: 588–600. https://doi.org/10.1038/s41380-018-0243-x PMID: 30283037

20. Ferland J-MN, Ellis RJ, Rompala G, Landry JA, Callens JE, Ly A, et al. Dose mediates the protracted effects of adolescent THC exposure on reward and stress reactivity in males relevant to perturbation of the basolateral amygdala transcriptome. Molecular Psychiatry. 2022. https://doi.org/10.1038/s41380-022-01467-0 PMID: 35236956

21. Jacobus J, Tapert SF. Effects of cannabis on the adolescent brain. Curr Pharm Des. 2014; 20: 2186–2193. https://doi.org/10.2174/13816128113199990426 PMID: 23829363

22. Miller M, Borges G, Orozco R, Mukamal K, Rimm EB, Benjet C, et al. Exposure to alcohol, drugs and tobacco and the risk of subsequent suicidality: Findings from the Mexican Adolescent Mental Health Survey. Drug and Alcohol Dependence. 2011; 113: 110–117. https://doi.org/10.1016/j.drugalcdep.2010.07.016 PMID: 20801585

23. Shalit N, Shoval G, Shlosberg D, Feingold D, Lev-Ran S. The association between cannabis use and suicidality among men and women: A population-based longitudinal study. Journal of Affective Disorders. 2016; 205: 216–224. https://doi.org/10.1016/j.jad.2016.07.010 PMID: 27449554

24. Wichstrøm L. Predictors of Adolescent Suicide Attempts: A Nationally Representative Longitudinal Study of Norwegian Adolescents. Journal of the American Academy of Child & Adolescent Psychiatry. 2000; 39: 603–610. https://doi.org/10.1097/00004583-200005000-00014 PMID: 10802978

25. Wong SS, Zhou B, Goebert D, Hishinuma ES. The risk of adolescent suicide across patterns of drug use: a nationally representative study of high school students in the United States from 1999 to 2009. Social Psychiatry and Psychiatric Epidemiology. 2013; 48: 1611–1620. https://doi.org/10.1007/s00127-013-0721-z PMID: 23744443

26. Kahn GD, Wilcox HC. Marijuana Use Is Associated With Suicidal Ideation and Behavior Among US Adolescents at Rates Similar to Tobacco and Alcohol. Arch Suicide Res. 2020/08/11 ed. 2022; 26: 520–533. https://doi.org/10.1080/13811118.2020.1804025 PMID: 32780674

27. Hinckley JD, Mikulich-Gilbertson SK, He J-P, Bhatia D, Ellingson JM, Vu BN, et al. Cannabis Use Is Associated With Depression Severity and Suicidality in the National Comorbidity Survey−Adolescent Supplement. JAACAP Open. 2023; 1: 24–35. https://doi.org/10.1016/j.jaacop.2023.02.002 PMID: 37538853

28. Sellers CM, Díaz-Valdés A, Oliver MM, Simon KM, O'Brien KHM. The relationship between alcohol and cannabis use with nonsuicidal self-injury among adolescent inpatients: Examining the 90 days prior to psychiatric hospitalization. Addict Behav. 2020/12/02 ed. 2021; 114: 106759–106759. https://doi.org/10.1016/j.addbeh.2020.106759 PMID: 33338906

29. Sellers CM, Diaz-Valdes Iriarte A, Wyman Battalen A, O'Brien KHM. Alcohol and marijuana use as daily predictors of suicide ideation and attempts among adolescents prior to psychiatric hospitalization. Psychiatry Res. 2019/02/03 ed. 2019; 273: 672–677. https://doi.org/10.1016/j.psychres.2019.02.006 PMID: 31207851

30. Randall K, Hayward K. Emergent Medical Illnesses Related to Cannabis Use. Mo Med. 2019; 116: 226–228. https://doi.org/10.1016/j.ejem.2013.12.013 PMID: 31527946

31. Jouanjus E, Leymarie F, Tubery M, Lapeyre-Mestre M. Cannabis-related hospitalizations: unexpected serious events identified through hospital databases. Br J Clin Pharmacol. 2011; 71: 758–765. https://doi.org/10.1111/j.1365-2125.2010.03897.x PMID: 21204913

32. Masonbrink AR, Richardson T, Hall M, Catley D, Wilson K. Trends in Adolescent Cannabis-Related Hospitalizations by State Legalization Laws, 2008–2019. Journal of Adolescent Health. 2021; 69: 999–1005. https://doi.org/10.1016/j.jadohealth.2021.07.028 PMID: 34511329
33. McBain RK, Cantor JH, Eberhart NK, Crowley C, Estrada-Darley I. Psychiatric and Substance Use Disorder Bed Capacity, Need, and Shortage Estimates in Sacramento County, California. Rand Health Q. 2022; 10: 1. PMID: 36484075
34. Agency for Healthcare Research and Quality, Rockville, MD. 2012. Healthcare Cost and Utilization Project (HCUP) US National Inpatient Sample Overview. 2012 [cited 21 Sep 2023]. Available: https://hcup-us.ahrq.gov/nisoverview.jsp.
35. Quarshie EN-B, Dey NEY, Oppong Asante K. Adolescent suicidal behaviour in Namibia: a cross-sectional study of prevalence and correlates among 3,152 school learners aged 12–17 years. BMC Psychiatry. 2023; 23: 169–169. https://doi.org/10.1186/s12888-023-04646-7 PMID: 36922818
36. Fresán A, Dionisio-García DM, González-Castro TB, Ramos-Méndez MÁ, Castillo-Avila RG, Tovilla-Zárate CA, et al. Cannabis smoking increases the risk of suicide ideation and suicide attempt in young individuals of 11–21 years: A systematic review and meta-analysis. Journal of Psychiatric Research. 2022; 153: 90–98. https://doi.org/10.1016/j.jpsychires.2022.06.053 PMID: 35810604
37. Fergusson DM, Boden JM, Horwood LJ. Cannabis use and other illicit drug use: testing the cannabis gateway hypothesis. Addiction. 2006; 101: 556–569. https://doi.org/10.1111/j.1360-0443.2005.01322.x PMID: 16548935
38. Han B, Compton WM, Einstein EB, Volkow ND. Associations of Suicidality Trends With Cannabis Use as a Function of Sex and Depression Status. JAMA Netw Open. 2021; 4: e2113025–e2113025. https://doi.org/10.1001/jamanetworkopen.2021.13025 PMID: 34156452
39. Stapinski LA, Montgomery AA, Araya R. Anxiety, depression and risk of cannabis use: Examining the internalising pathway to use among Chilean adolescents. Drug and Alcohol Dependence. 2016; 166: 109–115. https://doi.org/10.1016/j.drugalcdep.2016.06.032 PMID: 27427415
40. Patton GC, Coffey C, Carlin JB, Degenhardt L, Lynskey M, Hall W. Cannabis use and mental health in young people: cohort study. BMJ. 2002; 325: 1195–1198. https://doi.org/10.1136/bmj.325.7374.1195 PMID: 12446533
41. Gobbi G, Atkin T, Zytynski T, Wang S, Askari S, Boruff J, et al. Association of Cannabis Use in Adolescence and Risk of Depression, Anxiety, and Suicidality in Young Adulthood: A Systematic Review and Meta-analysis. JAMA Psychiatry. 2019; 76: 426–434. https://doi.org/10.1001/jamapsychiatry.2018.4500 PMID: 30758486
42. Hser Y-I, Mooney LJ, Huang D, Zhu Y, Tomko RL, McClure E, et al. Reductions in cannabis use are associated with improvements in anxiety, depression, and sleep quality, but not quality of life. J Subst Abuse Treat. 2017/07/29 ed. 2017; 81: 53–58. https://doi.org/10.1016/j.jsat.2017.07.012 PMID: 28847455
43. Feingold D, Weinstein A. Cannabis and Depression. Cannabinoids and Neuropsychiatric Disorders. 2020; 67–80. https://doi.org/10.1007/978-3-030-57369-0_5 PMID: 33332004
44. Flores MW, Granados S, Cook BL. US trends in the association of suicide ideation/behaviors with marijuana use among adolescents ages 12–17 and differences by gender and race/ethnicity. Front Psychiatry. 2023; 13: 1057784. https://doi.org/10.3389/fpsyt.2022.1057784 PMID: 36684023
45. Baiden P, LaBrenz CA, Asiedua-Baiden G, Muehlenkamp JJ. Examining the intersection of race/ethnicity and sexual orientation on suicidal ideation and suicide attempt among adolescents: Findings from the 2017 Youth Risk Behavior Survey. Journal of Psychiatric Research. 2020; 125: 13–20. https://doi.org/10.1016/j.jpsychires.2020.02.029 PMID: 32179279
46. Lindsey MA, Sheftall AH, Xiao Y, Joe S. Trends of Suicidal Behaviors Among High School Students in the United States: 1991–2017. Pediatrics. 2019; 144: e20191187. https://doi.org/10.1542/peds.2019-1187 PMID: 31611338
47. Molock SD, Boyd RC, Alvarez K, Cha C, Denton E-G, Glenn CR, et al. Culturally responsive assessment of suicidal thoughts and behaviors in youth of color. Am Psychol. 2023; 10.1037/amp0001140. https://doi.org/10.1037/amp0001140 PMID: 36913280
48. Rojas-Velasquez DA, Pluhar EI, Burns PA, Burton ET. Nonsuicidal Self-Injury Among African American and Hispanic Adolescents and Young Adults: a Systematic Review. Prev Sci. 2021; 22: 367–377. https://doi.org/10.1007/s11121-020-01147-x PMID: 32671672
49. Wu L-T, Zhu H, Mannelli P, Swartz MS. Prevalence and correlates of treatment utilization among adults with cannabis use disorder in the United States. Drug Alcohol Depend. 2017/05/29 ed. 2017; 177: 153–162. https://doi.org/10.1016/j.drugalcdep.2017.03.037 PMID: 28599214
50. Hasin DS, Saha TD, Kerridge BT, Goldstein RB, Chou SP, Zhang H, et al. Prevalence of Marijuana Use Disorders in the United States Between 2001–2002 and 2012–2013. JAMA Psychiatry. 2015; 72: 1235–1242. https://doi.org/10.1001/jamapsychiatry.2015.1858 PMID: 26502112

51. Lev-Ran S, Imtiaz S, Taylor BJ, Shield KD, Rehm J, Le Foll B. Gender differences in health-related quality of life among cannabis users: Results from the national epidemiologic survey on alcohol and related conditions. Drug and Alcohol Dependence. 2012; 123: 190–200. https://doi.org/10.1016/j.drugalcdep.2011.11.010 PMID: 22143039

52. Wilcox HC, Anthony JC. The development of suicide ideation and attempts: an epidemiologic study of first graders followed into young adulthood. Drug and Alcohol Dependence. 2004; 76: S53–S67. https://doi.org/10.1016/j.drugalcdep.2004.08.007 PMID: 15555817

53. Agrawal A, Nelson EC, Bucholz KK, Tillman R, Grucza RA, Statham DJ, et al. Major depressive disorder, suicidal thoughts and behaviours, and cannabis involvement in discordant twins: a retrospective cohort study. Lancet Psychiatry. 2017/07/24 ed. 2017; 4: 706–714. https://doi.org/10.1016/S2215-0366(17)30280-8 PMID: 28750823

54. Kelly LM, Drazdowski TK, Livingston NR, Zajac K. Demographic risk factors for co-occurring suicidality and cannabis use disorders: Findings from a nationally representative United States sample. Addict Behav. 2021/07/12 ed. 2021; 122: 107047–107047. https://doi.org/10.1016/j.addbeh.2021.107047 PMID: 34284313

55. Bresin K, Schoenleber M. Gender differences in the prevalence of nonsuicidal self-injury: A meta-analysis. Clinical Psychology Review. 2015; 38: 55–64. https://doi.org/10.1016/j.cpr.2015.02.009 PMID: 25795294

56. Nock M, Joinerjr T, Gordon K, Lloydrichardson E, Prinstein M. Non-suicidal self-injury among adolescents: Diagnostic correlates and relation to suicide attempts. Psychiatry Research. 2006; 144: 65–72. https://doi.org/10.1016/j.psychres.2006.05.010 PMID: 16887199

57. Grandclerc S, De Labrouhe D, Spodenkiewicz M, Lachal J, Moro M-R. Relations between Nonsuicidal Self-Injury and Suicidal Behavior in Adolescence: A Systematic Review. PLoS One. 2016; 11: e0153760–e0153760. https://doi.org/10.1371/journal.pone.0153760 PMID: 27089157

58. Kiekens G, Hasking P, Boyes M, Claes L, Mortier P, Auerbach RP, et al. The associations between non-suicidal self-injury and first onset suicidal thoughts and behaviors. Journal of Affective Disorders. 2018; 239: 171–179. https://doi.org/10.1016/j.jad.2018.06.033 PMID: 30014957

59. Hamza CA, Willoughby T. Nonsuicidal Self-Injury and Suicidal Risk Among Emerging Adults. Journal of Adolescent Health. 2016; 59: 411–415. https://doi.org/10.1016/j.jadohealth.2016.05.019 PMID: 27485906

60. Chesin MS, Galfavy H, Sonmez CC, Wong A, Oquendo MA, Mann JJ, et al. Nonsuicidal Self-Injury Is Predictive of Suicide Attempts Among Individuals with Mood Disorders. Suicide Life Threat Behav. 2017/02/17 ed. 2017; 47: 567–579. https://doi.org/10.1111/sltb.12331 PMID: 28211201
